# Special Issue “Natural Polymers and Biopolymers II”

**DOI:** 10.3390/molecules26010112

**Published:** 2020-12-29

**Authors:** Sylvain Caillol

**Affiliations:** ICGM, Univ Montpellier, CNRS, ENSCM, 34296 Montpellier, France; sylvain.caillol@enscm.fr

BioPolymers could be either natural polymers (polymer naturally occurring in Nature, such as cellulose or starch…), or biobased polymers that are artificially synthesized from natural resources. Since the late 1990s, the polymer industry has faced two serious problems: Global warming and the anticipation of limits in accessing fossil resources. One solution involves the use of sustainable resources instead of fossil-based resources. Hence, biomass feedstocks are a promising resource because of their sustainability. The current production of biopolymers is around 15 Mt/y, but biopolymers are one of the most dynamic polymer area. 

Natural polymers are materials that widely occur in nature, or are extracted from plants or animals. Some examples of natural polymers are proteins, cellulose, natural rubber, silk, and wool, starch or natural rubber.

Biobased polymers are synthesized from renewable resources (vegetal, animal or fungal) but it does not mean that they are biodegradable polymers. Hence, biodegradability is a special functionality conferred to a material, bio-based or not, and biobased sourcing does not entail biodegradability. Very recently, due the awareness of the volumes of plastic wastes, biodegradable polymers have gained increasing attention from the market and both scientific and industrial communities. 

This special issue of Molecules deals with the current scientific and industrial challenges of Natural and Biobased Polymers, through the access of new biobased monomers, improved thermo-mechanical properties, and by substitution of harmful substances.

Firstly, concerning the renewable resources, this issue proposes the use of innovative biobased derived from terpenes. Indeed, Nishida et al. reports a series of *exo*-methylene 6-membered ring conjugated dienes, which are directly or indirectly obtained from terpenoids, such as β-phellandrene, carvone, piperitone, and verbenone, were radically polymerized [1]. New terpene-based epoxy monomers were also synthesized by Couture et al. for the synthesis of high properties epoxy networks [2]. Additionally, Mora et al. reported the synthesis of vanillin-derived amines for the curing of epoxy thermosets [3]. Moreover, Della Vacche et al. studied the photocuring of cardanol-based monomers for biobased composites [4]. Photopolymerization was also studied on innovative eugenol-based methacrylates by Molina-Gutiérrez et al. for elaboration of biobased coatings [5]. Additionally, Montané et al. proposed an original mechanical study on the synthesis of humins-based epoxy resins [6]. Vegetable oils remain one of the most used resources for the synthesis of biobased polymers. Kohut et al. reported a feature article dedicated to plant oil-based monomers (POBM) in emulsion polymerization [7]. Hence, POBMs with different unsaturations in copolymerization reactions with conventional vinyl monomers allows for obtaining copolymers with enhanced hydrophobicity, provides a mechanism of internal plasticization and control of crosslinking degree. 

Then, natural polymers were also studied in order to improve or propose new properties. Hence, curcumin loaded biobased films were studied from alginate/cellulose/gelatin by Chiaoprakobkij et al. These films showed non-cytotoxicity to human keratinocytes and human gingival fibroblasts but interestingly exhibited potent anticancer activity in oral cancer cells [8]. Moreover, Shirosaki et al. reported chitosan microfibers with properties thanks to silane coupling agents [9]. Furthermore, Saremi et al. proposed a feature article on adhesion and stability of nanocellulose coatings on films and textiles [10]. Additionally, Bernal-Ballen et al. reported chitosan/PVA-based system with antibacterial activity against bacterial strains without adding a high antibiotic concentration [11]. The findings of this study suggest that the system may be effective against healthcare-associated infections, a promising view in the design of novel antimicrobial biomaterials potentially suitable for tissue engineering applications. Chitosan/PVA-based systems were also reported as a promising alternative for bone tissue regeneration by Pineda-Castillo et al. [12]. A feature article presenting new analytical strategy dedicated to multiscale structure of starches grafted with hydrophobic groups was also reported by Volant et al. [13]. Furthermore, Allegretti et al. studied the tuning of lignin characteristics by fractionation, based on solvent extraction and membrane-assisted ultrafiltration [14]. Rebiere et al. managed the characterization of non-derivatized cellulose by SEC in Tetrabutylammonium Fluoride/Dimethylsulfoxide [15]. Then, Klapiszewski et al. reported the preparation and comprehensive characterization of innovative additives to abrasive materials based on functional, pro-ecological lignin-alumina hybrid fillers [16]. Miscibility is also an important issue in biopolymer blends for analysis of the behaviour of polymer pairs through the detection of phase separation and improvement of the mechanical and physical properties of the blend. Ghaeli et al. reported a study dedicated to the formulation of a stable and one-phase mixture of collagen and regenerated silk fibroin (RSF), with the highest miscibility ratio between these two macromolecules [17]. Ghica et al. studied the development and optimization of some topical collagen-dextran sponges with flufenamic acid, designed to be potential dressings for burn wounds healing [18]. Bundled actin structures play an essential role in the mechanical response of the actin cytoskeleton in eukaryotic cells. Although responsible for crucial cellular processes, they are rarely investigated in comparison to single filaments and isotropic networks. Strehle et al. presented a new method to determine the bending stiffness of individual bundles, by measuring the decay of an actively induced oscillation [19]. Their experiments revealed that thin, depletion force-induced bundles behave as semiflexible polymers and obey the theoretical predictions determined by the wormlike chain model. 

Biobased polyurethanes (PUs) are a very dynamic class of polymers that are gaining increased attention in both academic and industrial communities. Hence, Carrico et al. proposed castor oil and glycerol based formulations for the synthesis of thermal insulation PU foams [20]. Moreover, Peyrton et al. reported an original kinectic study on the synthesis of oleo-based Pus [21]. Other polymers are also very attractive for scientific communities, such as poly(lactic acid) (PLA) and derivatives. Hence, Bensabeh et al. proposed the acrylate functionalization of butyl-lactate for the synthesis of biobased ABA copolymers which demonstrated competitive performance when compared with commercial pressure-sensitive tapes [22]. PLA was also studied for three-dimensional (3D) printing with cellulose nanofibrils by Wang et al. [23]. Additionally, Duchiron et al. proposed a feature article dedicated to the enzymatic synthesis of amino acids endcapped polycaprolactone in order to propose functional polyesters [24]. End of life of polymers are also a real challenge and reversible polymers are becoming more and more attractive. Hence, Durand et al. proposed an original article on bio-based thermo-reversible furan-based polycarbonate networks [25].

This themed issue can be considered as collection of highlights within the field of Natural Polymers and Biobased Polymers which clearly demonstrate the increased interest in this field. We hope that this will inspire researchers to further develop this area and thus contribute to futures more sustainable society.
